# Prediction of drug resistance profile of multidrug-resistant *Mycobacterium tuberculosis* (MDR-MTB) isolates from newly diagnosed case by whole genome sequencing (WGS): a study from a high tuberculosis burden country

**DOI:** 10.1186/s12879-022-07482-4

**Published:** 2022-05-27

**Authors:** Wenwen Sun, Xuwei Gui, Zheyuan Wu, Yangyi Zhang, Liping Yan

**Affiliations:** 1 Shanghai Clinical Research Center for Infectious Disease (Tuberculosis), Department of Tuberculosis and Shanghai Key Lab of Tuberculosis, Shanghai Pulmonary Hospital, School of Medicine, Tongji University, No. 507 Zhengmin Road, Shanghai, 200433 China; 2 Tuberculosis Prevention Division, Shanghai Center for Disease Control and Prevention, Shanghai, China

**Keywords:** Newly diagnosed multidrug resistant tuberculosis, Whole-genome sequencing, Minimal inhibitory concentration, Phenotypes Drug susceptibility test, Drug resistance gene mutation

## Abstract

**Objectives:**

Our aim was to assess the ability of the Whole-genome sequencing (WGS) in predicting drug resistance profile of multidrug-resistant mycobacterium tuberculosis (MDR-MTB) from newly diagnosed cases in China.

**Methods:**

We validated the Phenotypic drug Sensitivity Test (pDST) for 12 anti-tuberculosis drugs using the Bactec MGIT 960 system. We described the characteristics of the isolates enrolled and compared the pDST results with resistance profiles predicted by WGS.

**Results:**

The pDST showed that of the 43 isolates enrolled, 25.6% were sensitive to rifabutin (RFB); 97.7%、97.7%、93.0% and 93.0% were sensitive to cycloserine (Cs), amikacin/kanamycin (Ak/Km), para-aminosalicylic acid (Pas) and ethionamide Eto), respectively; 18.6% were resistant to fluoroquinolones (FQs) or second-line injections. Genotype DST determined by WGS of Ak/Km、Eto and RFP reached high consistency to 97.7% compared with pDST, followed by moxifloxacin (Mfx) 95.3%, levofloxaci (Lfx) and Pas 93%, streptomycin (Sm) 90.3%. The genotype DST of RFB and EMB showed low consistency with the pDST of 67.2 and 79.1%. WGS also detected 27.9% isolates of pyrazinamide(PZA)-related drug-resistant mutation. No mutations associated with linezolid (Lzd), bedaquiline (Bdq) and clofazimine (Cfz) were detectd.

**Conclusions:**

WGS has the potential to infer resistance profiles without time-consuming phenotypic methods, which could be provide a basis to formulate reasonable treatment in high TB burden areas.

**Supplementary Information:**

The online version contains supplementary material available at 10.1186/s12879-022-07482-4.

## Introduction

The emergence and spread of *Mycobacterium tuberculosis* (MTB) resistant to multiple anti-tuberculosis drugs is a global health concern. The inability to provide effective treatment options due to resistance to existing anti-TB drugs is a growing problem [1, 2]. China accounts for 14% of the world's multidrug-resistant *tuberculosis (*MDR-TB) patients [1]. Accurate and timely drug sensitivity test (DST) is the basis of effective treatment [3], which remains a challenge in the management of MDR-TB. Culture-based phenotypic DST (pDST) methods are currently the gold standard for the most commonly used methods for drug resistance detection, but the disadvantages are time consuming、need advanced laboratory infrastructure、qualified staff and strict quality assurance mechanism、inadequate detection of low level (culture-negative) resistant isolates and the lack of a uniform minimum inhibitory concentration (MIC) analysis/evaluation. For example, misclassification may be possible when CC is close to MIC [4]. Moreover, some drugs, such as pyrazinamide (PZA), may not be able to perform accurate pDST due to their growth characteristics [5]. The mechanism of drug resistance of MTB is mainly due to mutations of genes encoding drug targets or converting enzymes, including single nucleotide polymorphisms (SNPs)、insertions and deletions [6]. The advantages of genotypic methods include the ability to detect resistance quickly, but limitations remain in terms of accuracy (requiring reliable quality control) and relevance to treatment and clinical outcomes. Moreover, specific molecular methods could not cover all kinds of drugs [6–8]. Due to the multiple drugs required for treatment, corresponding gene loci need to be analyzed to predict the complete drug resistance profile. Recent studies supported that Whole—genome sequencing (WGS) could accurately predict drug resistance of MTB isolates [9–12]. A French study showed that WGS had a good predictive effect on drug resistance profiles of four first-line drugs and corrected the pDST results. WGS has also been recommended as an initial tool for routine practice in areas with low prevalence of TB [13]. However, such studies are still relatively limited in developing countries with high TB burdens.

In the present study, WGS was used to identify anti-TB drugs resistance gene mutations in preserved MDR-MTB isolates from newly diagnosed TB cases, and the results were compared with the pDST based on MGIT 960 culture and MIC assay. Our aim was to assess the ability of the WGS in prediction drug resistance profile of MDR-MTB isolates from newly diagnosed cases in China.

## Materials and methods

### Participants and specimen collection

From January 1, 2018 to December 30, 2020, We collected sputum or bronchoalveolar lavage fluid from newly diagnosed pulmonary tuberculosis (PTB) patients and performed mycobacterium culture and species identification using the BACTEC MGIT 960 system (Becton Dickinson Life Sciences, Inc., USA). The MTB positive isolates were preserved in strain library at the laboratory shanghai pulmonary hospital.

Isolates were included from newly diagnosed MDR-TB patients who had never received anti-TB treatment (ATT)or who had been on treatment for less than one month [14]. MDR-TB confirmed by pDST meaned at least resistant to Isoniazid (INH) and rifampicin (RFP),Extensively drug- resistant tuberculosis (XDR-TB) comfirmed by pDST meaned resistant to both fluoroquinolones (FQs)and second-line injection agents; pre-XDR comfirmed by pDST meaned resistant to either FQs or second-line injection agents. The definition of pre-XDR and XDR in the study were derived from pre-all-oral treatment period.

### MIC testing on MycoTB MIC plates

The MycoTB MIC plate, containing 12 lyophilized antibiotics: INH、RFP、rifabutin (RFB)、ethambutol (EMB)、moxifloxacin (Mfx), Levofloxacin (Lfx)、streptomycin (Sm)、amikacin (Am)、 kanamycin (Km)、para-aminosalicylic acid (Pas)、ethionamide (Eto) and cycloserine (Cs) with prepared concentrations [15]. MycoTB MIC plate tests were performed according to the instructions of the manufacturer.

The MIC breakpoints for each drug were as follows: 1.0 μg/mL for RFP, 0.2 μg/mL for INH, 0.5 μg/mL for RFB, 5.0 μg/mL for EMB, 2.0 μg/mL for Lfx, 0.5 μg/mL for Mfx, 4.0 μg/mL for Am, 25.0 μg/mL for Cs, 5.0 μg/mL for Eto, 5.0 μg/mL for Km, 2.0 μg/mL for PAS, and 2.0 μg/mL for Sm [16, 17]. When the MIC was equal to or lower than the CCs, isolates were defined as susceptible.

### Whole-genome sequencing

43 isolates included were revived on Lowenstein-Jensen media for amplification culture of 2 weeks and then collected in tubes containing TE buffer for DNA extraction. Extraction and purification of genomic DNA from clinical isolates was carried out following QIAamp DNA Mini Kit (QIAGEN, Valencia, CA) protocols [18]. Libraries were constructed by Nextera XT library prep (Illumina) and paired-end 150-bp DNA sequencing was performed on a Hiseq 2500 platform (Illumina). The WGS analysis, including mapping and SNP calling, was performed following a previously validated pipeline [19, 20]. The WGS of MTB H37Rv strain (GenBank accession number NC_000962.3) was used as the reference template for read mapping. Sequencing reads were mapped to the reference genome using Bowtie2 (v2.3.1) [21]. SAMtools (v1.6) was used for SNP calling with mapping quality greater than 30 [22].Fixed mutations (frequency ≥ 75%) were identified using VarScan (v2.3.6) with at least 10 reads supporting and the strand bias filter option on [23].We excluded all SNPs annotated in regions that were difficult to characterize with short read sequencing technologies, such as PPE/PE-PGRS family genes, phage sequence, insertion or mobile genetic elements [19, 20].

### Classification of lineages and sublineages

The classification of lineages and sublineages were identified from the clade-specific SNPs, based on the criteria included the SNP barcode for sublineage predictions reported previously [24, 25]

### Identification of drug resistance-associated variants

For drug resistance genetic assessment, single nucleotide polymorphisms (SNPs) data obtained by WGS were compared to the WHO catalogue of MTB complex mutations associated with drug resistance [26], seen in Table 1.

**Table 1:** Identification of drug resistance-associated variants:

Anti-Tuberculosis-drugs	Drug resistance-associated variants
Rfp	rpoB and rpoC
Sm	rrs and rpsl
INH	katG, inhA, oxyR-ahpC and fabG1 _promote
EMB	embB, embC, embA and embR
PZA	pncA, panD and rpsA
FQs	gyrA and gyrB
Am	rrs and tlyA
Km	eis_promoter
Eto	ethR, inhA, ethA and fabG1_promoter
Pas	thyA, folc and ribD
Lzd	rrl and rplC
Cfz	Rv0678
Bdq	Rv0678

### Statistical analysis

Statistical analysis was carried out using IBM SPSS Statistics version 22.0 software (IBM Corp., Armonk, NY, USA). The sensitivity、specificity, and accuracy (consistency) of WGS in predicting resistance to each drug were compared with pDST.

## Results

### Sequencing information

#### MIC results tested by MycoTB MIC plates

The MIC results of 43 isolates were summarized in Table 2. Among the included MDR-TB isolates from newly diagnosed cases, pDST indicated that 25.6% (11/43)isolates were sensitive to RFB; 79.1% (34/43) isolates were resistant to Sm; 30.2% (13/43) isolates were resistant to EMB. Most isolates were sensitive to Cs (97.7%, 42/43)、Pas (93.0%, 40/43) and Eto (93.0%, 40/43). 18.6% (8/43) isolates were resistant to Mfx, 16.3%(7/43) isolates were resistant to to Lfx, and most of them (n = 7) were resistant to both Mfx and Lfx.

**Table 2:** **a** The MIC results of 43 MDR-MTB isolates. **b** pDST resistance pattern of pre-XDR and XDR strains (n)

a												
	INH	RFP	RFB	EMB	Mfx	Ofx	Eto	Pas	Km	Ak	Sm	Cs
CCs (ug/ml)	0.2	1.0	0.5	5.0	0.5	2.0	5.0	2.0	5.0	4.0	2.0	25.0
1	4	16	2	4	1	4	10	0.5	2.5	0.5	32	16
2	4	16	0.5	32	0.5	2	1.2	0.5	1.2	0.25	32	32
3	4	16	4	1	0.25	0.5	1.2	0.5	1.2	0.12	8	8
4	4	2	1	8	0.25	1	1.2	8	2.5	0.25	16	8
5	4	2	1	4	0.25	1	5	8	2.5	0.25	16	8
6	2	16	2	1	0.12	0.5	0.6	0.5	1.2	0.12	0.5	2
7	2	16	4	8	4	16	2.5	0.5	1.2	0.25	32	8
8	2	16	4	4	0.06	0.25	0.6	0.5	1.2	0.12	32	8
9	4	2	0.12	2	0.12	0.5	0.6	0.5	2.5	0.25	32	16
10	4	16	2	8	0.12	0.25	1.2	0.5	2.5	0.5	8	16
11	4	16	4	8	0.06	0.25	1.2	0.5	1.2	0.25	32	8
12	4	16	0.25	4	0.12	0.5	1.2	0.5	2.5	0.25	32	16
13	2	4	0.25	8	0.12	2	0.6	0.5	0.6	0.12	32	8
14	2	16	4	8	1	4	1.2	0.5	5	0.5	32	16
15	1	8	16	4	0.06	0.25	0.3	0.5	1.2	0.12	0.25	8
16	2	2	0.12	2	0.12	0.25	1.2	0.5	1.2	0.25	0.25	16
17	4	16	8	8	0.06	0.25	0.6	0.5	1.2	0.25	32	16
18	4	16	4	4	0.12	0.5	1.2	1	1.2	0.25	32	16
19	1	16	4	8	0.12	0.25	0.3	0.5	1.2	0.25	32	16
20	0.4	16	1	8	0.12	0.25	10	2	2.5	0.25	0.25	16
21	4	16	4	1	0.12	0.25	1.2	0.5	1.2	0.25	32	8
22	1	16	1	2	0.06	0.25	0.6	0.5	2.5	0.25	0.5	8
23	0.4	16	8	1	0.12	0.5	0.6	16	1.2	0.25	32	16
24	4	16	2	8	4	8	2.5	0.5	1.2	0.25	32	16
25	2	8	0.25	2	0.12	0.5	0.6	0.5	1.2	0.25	0.5	16
26	4	16	1	4	0.12	0.5	0.6	0.5	2.5	0.25	32	8
27	2	4	0.25	8	0.06	0.25	0.6	0.5	1.2	0.25	8	4
28	4	16	4	8	0.12	0.5	0.6	0.5	0.6	0.12	32	4
29	0.4	16	4	2	1	2	20	0.5	0.6	0.25	4	2
30	2	16	4	1	0.12	0.25	1.2	0.5	2.5	0.12	0.5	4
31	2	16	0.5	4	0.12	0.25	1.2	0.5	1.2	0.25	32	16
32	2	16	8	1	0.12	0.5	1.2	0.5	1.2	0.25	32	4
33	1	16	2	0.5	0.06	0.25	0.3	0.5	0.6	0.12	0.5	2
34	2	16	16	4	4	8	2.5	0.5	1.2	0.25	16	4
35	2	16	4	2	0.06	0.25	0.6	1	0.6	0.25	2	4
36	1	16	16	4	0.12	0.25	0.6	1	0.6	0.12	32	8
37	2	16	16	4	0.06	0.25	0.3	0.5	0.6	0.12	0.5	2
38	4	16	0.25	8	1	4	0.6	0.5	40	16	32	16
39	4	16	4	4	0.12	0.5	0.6	0.5	2.5	0.25	32	16
40	2	2	0.12	2	0.12	0.5	2.5	0.5	1.2	0.25	32	16
41	1	8	0.12	1	0.06	0.25	2.5	0.5	0.6	0.12	0.25	8
42	1	16	2	4	0.06	0.25	0.3	0.5	1.2	0.12	0.25	2
43	0.4	16	8	4	8	16	5	0.5	1.2	0.25	32	8
Drug-resistant isolates(n)	43	43	32	13	8	7	3	3	1	1	9	1

b					
	No. of strains	Mfx	Lfx	Km	Ak
Pre-XDR	1	R	S	S	S
	6	R	R	S	S
	0	S	R	S	S
XDR	1	R	R	R	R

*CCs* critical concentrations

*XDR* Extensively drug- resistant tuberculosis

#### Gene mutations associated with drug resistance

The sequencing quality control results of the isolates showed that the average sequencing depth of the genome was from 79.5 to 223.6 (mean:148.1), the genome 10X coverage was from 98.2 to 99. 5 (mean:98.9), and the genome 1X coverage was from 98.9 to 99.9 (mean:99.4). The results of WGS were interpretable (depth coverage ≥ 30x) for all MTB isolates.

#### Genotypic resistance of 43 phenotypic MDR-MTB isolates to first-line anti-tuberculosis drugs

A total of 43 MDR isolates were enrolled. We found that most (90.7%, 39/43) of the isolates were lineage 2 (L2)/Beijing ( n = 39), followed by lineage 4 (L4)/European and American type (n = 4). Most(84.6%, 33/39)of the Beijing type were found to be modern Beijing type by sub-lineages analysis and the rest (15.4%, 6/39) were ancestral Beijing strains. (seen in Additional file 1).

Table 3 lists all of the mutations associated with first-line anti-TB drugs of the enrolled isolates identified by WGS. Except for 1 strain (MIC = 16 g/mL), all other MDR-MTB isolates (97.7%) had drug-resistant determinant mutations in ropb gene, among which S450L was the most common mutation, which was found in 23 (54.8%) isolates. Meanwhile, 8 isolates (19.0%) had 2 mutations. Of the 32 Rfb resistant isolates, 31(98.9%)isolates showed resistance-determinant mutations in rpoB gene. In 11 Rfb-sensitive isolates, ropb gene mutations were also detected in all of them (100%). In 43 MDR-MTB resistant isolates, 37 isolates (86.0%) with INH resistance- determinant mutations all carried catalase-peroxidase enzyme (katG) S315T mutation. Among the 14 EMB resistant isolates, 13 EMB-resistant related mutant isolates (92.9%) carried the resistance- determinant mutation of embB, among which 6 (46.15%) were M306V mutants and 3 (23.1%) were M306L mutants. Of the 29 EMB sensitive isolates, 8 (27.6%) also carried EMB resistance -determinant mutations: 7 carried the embB mutations and 1 carried the embA mutation. Thirty -one of the 34 Sm resistant isolates (91.2%) had resistance-determinant mutations, 25 (80.6%) were rspl gene mutations, 3 (9.7%) were rrs gene mutations、3 (9.7%) were rspl and rrs mutations. The rpsl K43R was the most common type of gene mutation that was found in 20 Sm resistant stanis (64.5%).

**Table 3:** Genotypic resistance of 43 phenotypic MDR strains to first-line anti-TB drugs

Drug	Resistance phenotype (No. of strains)	Resistance genotype			MIC (µg/mL)	Total
		Gene	Mutations	No. of strains		
RFP	Resistant (43)	*rpoB*	S450L	23	16	42/43
			D435V	3	16	
			D435G	1	16	
			Q450L	1	2	
			L452P	1	2	
			H445Y	4	16	
			Q432P	1	16	
			H445Q\S450W	1	16	
			L430R\H445Y	1	16	
			D435Y\P454L	1	1	
			L430R\D435N	1	1	
			N437D\L452P	1	16	
			L430P\S431G	1	16	
			A286V\S450L	1	0.12	
			L430R\D435G	1	8	
RFB	Resistant (32)	*rpoB*	S450L	23	2,4,8,16	31/32
			Q450L	1	2	
			L452P	1	1	
			H445Y	3	2,16	
			H445Q\S450W	1	2	
			L430R\H445Y	1	4	
			N437D\L452P	1	1	
	Susceptible (11)	*rpoB*	D435G	1	0.5	11/11
			D435V	3	0.25	
			S450L	2	0.12, 0.25	
			Q432P	1	0.12	
			D435Y\P454L	1	0.12	
			L430R\D435N	1	0.25	
			A286V\S450L	1	0.12	
			L430R\D435G	1	0.12	
INH	Resistant (43)	*katG*	S315T	37	1, 2, 4	37/43
EMB	Resistant (14)	*embB*	Q497R	1	32	13/14
			G406A	1	8	
			M306V	6	8	
			M306L	3	8	
			E405D	1	8	
			Q497K	1	8	
	Susceptible (29)	*embB*	G406A	1	4	8/29
			M306V	2	4	
			M306L	4	2, 4	
		*embA*	C 12 T	1	4	
Sm	Resistant ( 34)	*rpsL*	K43R	20	8, 32	31/34
			K88R	5	8, 16, 32	
		*rrs*	A514C	2	8,32	
			C517T	1	32	
		*rpsl/rrs*	K43T/A514C	2	32	
		*rpsl/rrs*	K43R/A1401G	1	32	
	Susceptible (9)	*rrs*	A514C	1	2	1/9

#### Genotypic resistance of 43 phenotypic MDR isolates to second-line anti-TB drugs

Of the 7 Lfx resistant isolates, 5 (71.4%) had gyra mutations, while among the 36 Lfx sensitive isolates, 1 showed gyra mutation (2.0%). Of the 8 Mfx resistant isolates, 5 (62.5%) showed gyra mutations and 1 showed gyrb mutation ( 12.5%). However, among the 35 Mfx sensitive isolates, 1 showed gyra mutation (2.9%). The rrs mutation occurred in 1 case of Ak and Km resistance, in which the strain had both Lfx and Mfx resistance-determinant mutations. However, rrs mutation also occurred in 1 of the 42 Ak/Km sensitive cases (2.38%). The fabg1 mutation occurred in all 3 (100%) Eto resistant isolates and also occurred in 1 (2.5%) of the 40 Eto sensitive cases. The folc mutation occurred in 1 of the 3 Pas (33.3%) resistant isolates. Of the 40 pas sensitive cases, 1 folc mutation also occurred (2.5%). (Table 4).

**Table 4:** Genotypic resistance of 43 phenotypic MDR strains to second-line anti-TB drugs

Drug	Resistance phenotype (No. of strains)	Resistance genotype			MIC (µg/mL)	Total
		Gene	Mutations	No. of strains		
LFX	Resistant (7)	*gyrA*	A90V	2	4	5/7
			D94A	1	16	
			D94G	1	16	
			D94H	1	8	
	Susceptible (36)	*gyrA*	D94G	1	0.5	1/36
MFX	Resistant (8)	*gyrA*	A90V	2	1	6/8
			D94A	1	1	
			D94G	1	4	
			D94H	1	4	
		*gyrb*	S447F	1	1	1/8
	Susceptible (35)	*gyrA*	D94G	1	0.12	1/35
Ak	Resistant (1)	*rrs*	A1401G	1	16	1/1
	Susceptible (42)	*rrs*	C517T	1	0.25	1/42
Km	Resistant (1)	*rrs*	A1401G	1	40	1/1
	Susceptible (42)	*rrs*	C517T	1	1.2	1/42
Eto	Resistant (3)	*fabG1-inhA*	C8T	1	10	3/3
			C15C	2	20	
	Susceptible (40)	*fabG1-inhA*	C15C	1	5	1/40
Pas	Resistant (3)	*folC*	143 T	1	16	1/3
	Susceptible (40)	*folC*	S150G	1	1	1/40

WGS also detected 12 isolates of PZA-related drug-resistant gene mutation pncA (27.9%, 12/43). No mutations were seen in genes encoding for Lzd、Cfz and Bdq.

#### Comparison of WGS and pDST in drug resistance detection

To assess the prediction ability of drug resistance in WGS, we calculated the consistency between WGS and MIC-pDST for 43 isolates in 12 anti-TB drugs (Table 5). In MDR-MTB isolates from newly diagnosed cases in China, the pDST of Rfb showed the lowest consistent with its genotype (67.2%), the differences were that rifamycin resistance determinant mutations were found in all MIC Rfb sensitive isolates; genotype DST determined by WGS of Ak、Km、Eto and Rfp reached high consistency to 97.7% compared with MIC, followed by Mfx ( 95.3%)、Lfx and Pas (both 93%) and Sm (90.3%). The consistency between INH resistance genotype and pDST was relatively poor (86%), all 6 cases of inconsistencies occurred were pDST resistance but no INH resistance determinant mutations were identified. The genotypic and phenotypic DST of EMB showed the second lowest consistency (79.1%), with most (88.89%) due to the EMB resistance determinant mutations (7 embB, 1embA) detected in pDST susceptibility isolates.

**Table 5:** Sensitivity, specificity, and overall accuracy of WGS compared to MIC in MDR-TB strains from newly-diagnosed cases: (%)

	Sensitivity	Specificity	Accuracy (consistency)
INH	86.0	–	86.0
RFP	97.7	–	97.7
RFB	96.9	0	67.2
Sm	91.2	88.9	90.7
FQs	75	97.1	93.0
Lfx	71.4	97.2	93.0
Mfx	87.5	97.1	95.3
EMB	92.9	72.4	79.1
Ak	100	97.6	97.7
Km	100	97.6	97.7
Eto	100	97.5	97.7
Pas	33.3	97.5	93.0

## Discussion

The present study found that the MDR-MTB isolates from newly diagnosed cases in China had following characteristics in pDST: the majority were also resistant to Rfb and Sm. Most of the isolates were sensitive to Eto、Pas、CS and second-line injections. Notably, most FQS-resistant isolates were resistant to both levofloxacin and moxifloxacin. FQS, a class A drug recommended by the latest WHO guidelines for the treatment of MDR-TB [27], should be selected with strict reference to DST in the protocol formulation of newly-diagnosed MDR-TB patients in China.

Although the latest guideline no longer recommend second-line injections due to their significant adverse effects [27], however, the results of the study showed that the resistance rate of MDR-TB isolates from newly diagnosed cases to the drugs was low.

It has been shown that the genotype and polymorphism of MTBC infection can affect the CCs of anti-tuberculosis drugs. WGS on clinical isolates may increases the possibility of identifying strain resistance on the basis of MIC and may guide effective treatment within days [28]. In the study, 43 MDR-MTB isolates identified by MIC were tested by WGS for drug resistance gene spectrum, and the results were as follows: deterministic mutation of rpoB gene was found in 97.7% of RFP resistant isolates, 54.8% of which were S450L mutations,98.9% of RFB resistant isolates also had rpoB mutations. However, we found that 11 RFB-sensitive isolates also detected rpoB gene mutations. RFB is a semi-synthetic derivative of rifamutin S, which together with RFP is part of the rifamutin family [29]. Although cross-resistance to RFP and RFB is common, RFB-sensitive/RFP resistant isolates have been reported, and RFB has been suggested as a viable alternative for the treatment of MDR-TB associated with specific rpoB mutations [30]. Previous study has found that the proportion of RFB-sensitive/RFP resistant isolates is between 13–28% [31]. In the present study, we included isolates from newly diagnosed MDR-TB patients, we found that D435G, D435V、S450L and other residues (Table 2) may be related to RFB-sensitive/RFP resistant pattern. The proportion of RFB-sensitive/RFP resistant isolates in all rpoB mutations was similar to that in previous study (26.2%, 11/42, Table1). In general, the rpoB mutant gene has a good predictive value for RFP resistance, while it may still be impossible to accurately predict RFB resistance. Our study identified a proportion of RFB-sensitive/RFP resistant isolates from newly diagnosed MDR-TB patients, so RFB may be a good treatment option for MDR-TB associated with specific rpoB mutations. Our findings need to be validated in larger studies, and if confirmed, rpoB gene sequencing may be recommended for all suspected MDR isolates in order to quickly determine whether RFB is a possible treatment option for the patients. The molecular basis of INH resistance is mediated by mutations in the promoter region of katG or inhA genes. The most common resistance mechanism was identified as the katG S315T mutation. This mechanism is related to the high level of INH resistance in MDR isolates [32]. Previous studies have reported that mutations in the regulatory and coding regions of inhA lead to high concentration resistance to INH and cross-resistance to Eto [33]. In the study, 37 isolates (86.0%) with INH resistance- determinant mutations all carried katGS315T mutation. No INH related gene mutations were found in the other 6 phenotypic drug-resistant isolates. Our study shows that katG could be a good prediction gene for INH resistance, but phenotypic resistance MDR-TB isolates may not be detected by gene mutation. The PZA pDST is technically challenging and unreliable due to the acidic pH required for culture [34]. Mutations in the pncA gene and its promoter region continue to be the most common mechanism for mediating PZA resistance. A multi-country study has reported a strong link between PZA and Rfp resistance, confirming the role of predicted PZA resistance in MDR-TB [5]. In the present study, 27.9% of the newly-diagnosed MDR isolates had the PZA determinant drug resistance gene mutation pncA. Based on the reliability of pncA gene mutations described in the literature, we proposed to predict PZA susceptibility based on these mutations in order to improve therapeutic efficacy. Resistance to EMB is mainly mediated by mutations in the embB [35]. In the present study, 13 of the 14 EMB-resistant isolates (92.9%) carried embB mutations, while 8 of the 29 EMB-sensitive isolates (27.6%) also carried EMB -resistance mutations (7 for embB mutations and 1 for embA mutations). The results suggested that the sensitivity of EMB resistance gene mutation in newly-diagnosed MDR-MTB isolates was higher than the existing study [36], but the occurrence of mutation may not predict drug resistance reliably. On the other hand, previous study has shown that the CCs of EMB pDST defined by the WHO may be too high (5ug/ml), which may lead to inappropriate treatment due to the assumed EMB susceptibility [13]. The conclusion may consistent with our results to a certain extent that some isolates with EMB-resistant mutation and MIC sensitivity may still be EMB-resistant. The main mechanism of Sm resistance is mediated by mutations in the rpsl and rrs genes [37]. 91.2% of Sm-resistant isolates had drug-resistant gene mutation and rpsl K43R was the most common type of gene mutation (64.5%). The rrs mutation was also found in 1 Sm-sensitive isolate. Aminoglycosides (km and Ak) are second-line injectable agents currently used in the treatment of MDR-TB. High levels of resistance are associated with mutations in the rrs gene [38]. In the present study, the rrs mutation occurred in only 1 case of Ak and Km resistance which was consistent with the MIC result. FQs are currently one of the core drugs recommended by WHO for the treatment of MDR-TB [27]. Resistance to FQs was mainly associated with mutations in the gyrA and gyrB genes [39]. Among the FQx resistant isolates in the study, gyra was the most common mutation, Only 1 stain of gyrb mutation showed phenotypic resistance to Mfx. Our study suggested that most of the resistance mutations associated with FQS were related to pDST, with only 1 mutation occurring in the susceptible strain. However, there were also 2 FQs phenotypic resistance without genetic mutations. So the overall consistency was 93.0%. The main mechanism that mediates resistance to pas is mutation of thya gene [40]. However, in the present study, only 1 of the 3 pas resistant isolate (33.3%) developed folc mutation and 1 of the 40 pas sensitive isolate (2.5%) also developed folc mutation. Our study suggests that the resistance rate of pas in newly-diagnosed MDR-MTB isolates could be relatively low in China, the predictive value of genotype may be limited, which is still to be studied. The fabg1 mutation occurred in all Eto resistant isolates and in 2.5% of the 40 Eto sensitive cases. Study from Iran showed that about 54% of RFP-resistant isolates were identified as Eto resistant [41]. But In the present study, the Eto resistance rate of newly-diagnosed MDR-TB cases in China was low (7.0%). The sensitivity and specificity of Eto resistance gene detection were 100% and 97.5%, respectively. Previous study also suggested that most of Eto resistant isolates were cross-resistant to INH related to inhA mutation. However, in the study, we found that the INH resistant mutation were all sited in katG, suggesting that Eto may be a good alternative drug for newly-diagnosed MDR-PTB in China. Finally, we found that the LZD、CFZ and Bdq had no drug-resistant mutations in the isolates. No phenotypic resistance was found for Cs. The results suggested that these drugs may be good candidates for newly-diagnosed MDR-TB in China.

In summary, WGS has high predictive power to infer resistance profiles without the need for time-consuming pDST and having the potential to determine resistance to most drugs through a single analysis [42]. However, there are still some inconsistencies between phenotype and genotype DST, especially in EMB、Rfb and Pas. We suggest that pDST could be complementary to WGS to identify non-highly credible mutations in antituberculous drug-related loci (e.g., INH、Pas、RFB), and ultimately confirm the drug susceptibility of MTB isolates,WGS could also be used to supplement the prediction of drug resistance to drugs for which phenotypic susceptibility may be less reliable (e.g., Cs、PZA、EMB). A significant proportion of newly diagnosed MDR-TB cases in China have FQs resistance, and WGS can be used to identify resistant mutations at an early stage to reduce ineffective treatment.

## Supplementary Information

**Additional file 1.** The original sequencing data.

## Data Availability

The WGS data are available in the NCBI SRA database, under accession number PRJNA804712 (https://www.ncbi.nlm.nih.gov/sra/PRJNA804712).
